# Research Progress and Outlook of Molecular Dynamics Simulation on Carbon Dioxide Applied for Methane Exploitation from Hydrates

**DOI:** 10.3390/molecules29235579

**Published:** 2024-11-26

**Authors:** Qiannan Yu, Chenglong Li, Boyang Peng, Huimin Tang, Tao Yang, Yang Yu, Kun Zhang, Zhijing Chen

**Affiliations:** 1 College of Energy and Power Engineering, Guangdong University of Petrochemical Technology, Maoming 525000, China; yuqn@gdupt.edu.cn (Q.Y.); lamb_tao@outlook.com (T.Y.); pecewe@outlook.com (Y.Y.); zkgdupt@outlook.com (K.Z.); 2 Exploration and Development Research Institute, Daqing Oilfield Co., Ltd., Daqing 163712, China; 3 Geological Research Institute of No. 4 Oil Production Plant, Daqing Oilfield Co., Ltd., Daqing 163511, China; eclipsedelphi@126.com; 4 Hainan Branch CNOOC (China) Co., Ltd., Haikou 570311, China; tanghm2@cnooc.com.cn

**Keywords:** natural gas hydrate, carbon dioxide replacement method, molecular dynamics simulation, methane exploitation

## Abstract

Research progress of carbon dioxide applied for methane exploitation from hydrates is summarized, with a focus on advances in molecular dynamics simulations and their application in understanding the mechanism of carbon dioxide replacement for hydrate exploitation. The potential of carbon dioxide in enhancing energy recovery efficiency and promoting carbon capture and storage is emphasized. An overview is provided of the advancements made in utilizing carbon dioxide for methane hydrate exploitation, highlighting its significance. Subsequently, the theoretical foundations and techniques of molecular dynamics simulations are delved into, encompassing key elements such as statistical ensembles, molecular force fields, and numerical solution methods. Through simulations, various characterization parameters including mean square displacement, radial distribution functions, coordination numbers, angular order parameters, and hydrogen bonds are computed and analyzed, which are crucial for understanding the dynamic changes in hydrate structures and the replacement process. Thorough research and analysis have been conducted on the two possible and widely debated mechanisms involved in the replacement of methane hydrates by carbon dioxide, with a particular emphasis on guest molecular replacement and hydrate reconfiguration. These processes encompass the intricate interactions between carbon dioxide molecules and the cage-like structure of hydrates, as well as the rearrangement and stabilization of hydrate structures. Several key issues surrounding the application of carbon dioxide for methane hydrate exploitation are identified, including the influence of thermodynamic conditions, the selection of auxiliary gases, and other potential factors such as geological conditions and fluid properties. Addressing these issues is crucial for optimizing the extraction process and enhancing economic and environmental benefits. A theoretical foundation and technical reference for the application of carbon dioxide in methane hydrate exploitation are provided, while future research directions and priorities are also outlined.

## 1. Introduction

In order to achieve the goal of ‘peak carbon and carbon neutrality’, adjusting the energy mix to reduce carbon emissions has become a key task. At present, the global energy supply mainly relies on traditional fossil fuels such as coal and oil. As an efficient and clean future energy source, the development of natural gas hydrates is of great significance for improving the energy structure and protecting the environment. However, natural gas hydrates can only be stabilized under specific temperature and pressure conditions, and their extraction process can easily become uncontrollable, leading to methane leakage and geological disasters. The carbon dioxide replacement method has received widespread attention due to its significant carbon sequestration effect and high geological stability. This method involves replacing methane in natural gas hydrates with carbon dioxide to form more stable carbon dioxide hydrates, thereby reducing the risk of geohazards and facilitating carbon capture and utilization.

**Figure 1:**
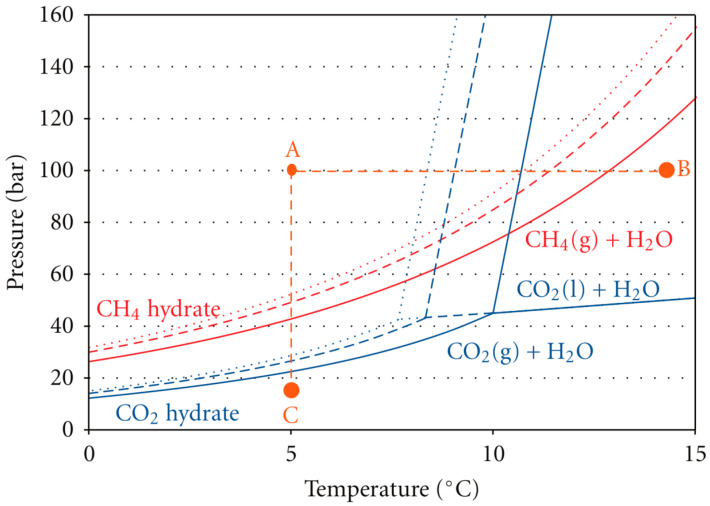
Equilibrium curves of methane hydrate and carbon dioxide hydrates for different salinities.

Under specific temperature and pressure conditions, methane hydrates decompose while carbon dioxide hydrates remain stable. Based on this property, Ebinuma, T. [1] and Ohgaki, K. et al. [2] proposed the carbon dioxide replacement method in the mid-1990s. This method, by injecting carbon dioxide into the hydrate reservoir, induces the decomposition of methane hydrate and releases methane, while generating a more stable carbon dioxide hydrate. The heat released during this process helps to maintain the continued decomposition of the methane hydrate. In 2011, the carbon dioxide displacement method was applied for a gas hydrate production test in the Ignik Sikumi 1 well in Prudhoe Bay on the North Slope of Alaska in the United States [3]. In the same year, carbon dioxide emissions from factories were injected into subsea gas hydrate reservoirs to extract natural gas in the SUGAR (SUbmarine GAs hydrate Reservoirs) project in Germany [4]. Sloan, E.D. et al. [5] developed a phase equilibrium diagram for the methane–carbon dioxide–water system based on the phase equilibrium characteristics of methane hydrate and carbon dioxide hydrate; Janicki, G. et al. [6] developed equilibrium curves of methane hydrate and carbon dioxide hydrates for different salinities, further supporting the mechanism of the carbon dioxide replacement method (Figure 1).

The figure shows that the phase equilibrium pressure of carbon dioxide hydrate is lower than that of methane hydrate under most temperature conditions, while the phase equilibrium temperature of carbon dioxide hydrate is higher than that of methane hydrate under most pressure conditions. This suggests that carbon dioxide hydrates are more stable than methane hydrates, which exist under relatively relaxed conditions and are thermodynamically less prone to decomposition. The studies of Svandal, A. et al. [7] have further confirmed that carbon dioxide hydrates are thermodynamically more stable than methane hydrates over a specific range of temperatures and pressures. Uchida, T. et al. [8] observed by gas chromatography and Raman spectroscopy that the phase equilibrium pressure of carbon dioxide hydrate is lower than that of methane hydrate, indicating that the thermodynamic stability of carbon dioxide hydrate is higher under certain conditions. The same conclusion was obtained by Akihiro, H. [9] in the determination of phase equilibrium temperatures and pressures of subfreezing carbon dioxide and methane hydrate. Ota, M. et al. [10] investigated the process of carbon dioxide substitution for methane hydrate at 3.6 MPa and 273.2 K, studied the replacement of methane hydrate by carbon dioxide, and showed that the ratio of methane hydrate decomposition to carbon dioxide hydrate production was greater than 1. Yoon, J.H. et al. [11] studied the conversion of methane hydrate to carbon dioxide hydrate using in situ Raman spectroscopy and found that enhancement of the aqueous phase promoted the diffusion of methane and carbon dioxide, allowing the reaction to proceed rapidly. Ota, M. et al. [12] developed a kinetic model for the decomposition of methane hydrate and the formation of carbon dioxide hydrate. Yezdimer, E.M. et al. [13] demonstrated through MDS that the Gibbs free energy during carbon dioxide substitution is negative, proving that the method is thermodynamically feasible. Experimental studies by Goel, N. [14] also showed that, in a specific range of temperatures and pressures, carbon dioxide hydrates can be stabilized, while methane hydrates are difficult to form.

Li, Z.Z. et al. [15] have shown that temperature and pressure have a significant effect on the replacement of natural gas hydrate by carbon dioxide, and higher temperature and pressure are more favorable for the replacement reaction. Park, Y. et al. [16] found that the addition of an auxiliary gas, such as nitrogen, significantly improved the replacement efficiency compared with pure carbon dioxide gas, and Pandey, J.S. et al. [17] confirmed that the replacement efficiency of methane increased with the increase in the carbon dioxide concentration in the gas mixture and that the carbon dioxide in the gas mixture made the hydrate more stable. In addition, replacement using a gas mixture of hydrogen and carbon dioxide is more efficient than a mixture of nitrogen and carbon dioxide. Hydrogen molecules are smaller and easy to diffuse in the hydrate, which reduces the partial pressure of methane in the gas phase and helps to destabilize the methane hydrate and promote its decomposition [18]. The study by Ding, Y.L. et al. [19] showed that the replacement efficiency of a mixture of hydrogen and carbon dioxide was about 20% higher than that of pure carbon dioxide under the same conditions. Studies by Sun, Y.F. et al. [20] and others further showed that the decomposition rate of methane hydrate was accelerated with the increase in hydrogen content in the gas mixture, and the replacement rate of methane could be more than 90% under suitable temperature and pressure conditions [21].

**Figure 2:**
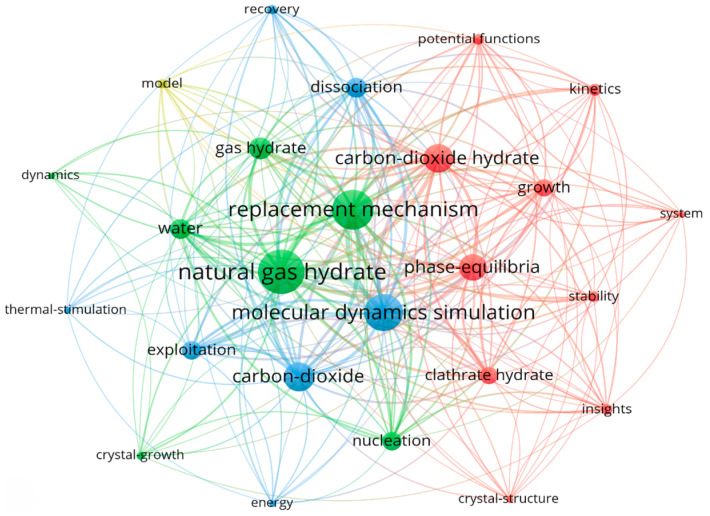
High-frequency keyword clustering co-occurrence map of natural gas hydrates.

Molecular dynamics simulation is an advanced computational tool that provides a key means of investigating the use of carbon dioxide in natural gas hydrate extraction. It offers greater flexibility and lower cost than traditional experimental methods, enabling in-depth exploration of alternative mechanisms, optimizing efficiency, and accelerating the commercial application of the technology. Based on the Web of Science core database, the literature related to molecular dynamics simulation of carbon dioxide displacement method was collected between 2000 and 2024. To ensure the relevance and accuracy of the data, the literature unrelated to the research topic was manually screened and excluded. Through metrological analysis, the current status, hotspots, and development trends of molecular dynamics simulation in the research of carbon dioxide replacement methods are objectively presented. The keyword clustering co-occurrence map is shown in Figure 2, which reveals the research theme and development lineage in this field and provides the idea of literature research for the progress of carbon dioxide applied for methane exploitation from hydrates. Based on the high-frequency keyword clustering co-occurrence map of natural gas hydrates, the primary focus, challenges, and key research areas in molecular dynamics simulation on carbon dioxide replacement for methane hydrates have been identified. The fundamentals of molecular dynamics simulation are first outlined, followed by a discussion of the main simulation techniques and characterization parameters specific to carbon dioxide application in methane hydrate extraction. Subsequently, an analysis of existing research on the mechanisms of carbon dioxide replacement in hydrates is presented. The study concludes with insights into the impact of thermodynamic conditions, auxiliary gases, and other factors on replacement rate and efficiency, as determined through molecular dynamics simulation studies.

## 2. Fundamentals of Molecular Dynamics Simulation

Molecular dynamics simulation (MDS) is a computational method based on the principles of Newtonian mechanics for modeling the motion and interactions of molecular systems. By calculating the forces between atoms, MDS is able to depict the trajectories and energy changes of molecular systems. In 1957, Alder, B.J. and Wainwright, T.E. [22] first used MDS to study the microscopic motions of 32 to 500 rigid spheres, initiating the use of MDS to study the macroscopic properties of matter. Subsequently, Rahman, A. [23] simulated the molecular dynamics of liquids using the Lennard–Jones potential function model in 1964. In 1967, Verlet proposed the widely used Verlet’s algorithm [24], which calculates the velocity, acceleration, and position of particles step by step and greatly promoted the development of MDS. As an important branch of molecular simulation methods, MDS is based on the principles of quantum mechanics and statistical mechanics and takes advantage of the advances in computer science to provide a new way to study microscopic phenomena. Especially for systems such as natural gas hydrates, whose microscopic properties are difficult to directly observe in experiments, MDS shows unique advantages.

### 2.1. Theoretical Basis

The core of MDS is the solution of the integral of the equations of motion of classical mechanics. Based on classical mechanics, it is known that the force on an atom is equal to its potential energy gradient in the *x*, *y*, and *z* directions, which leads to the equation that the force on any atom *i* in the system is equal to the potential energy gradient [25].

(1) $$\vec{F_{i}}=-\nabla_{i}U=-\left(\vec{i}\frac{\partial }{\partial x_{i}}+\vec{j}\frac{\partial }{\partial y_{i}}+\vec{k}\frac{\partial }{\partial z_{i}}\right)U$$

Combining Newton’s laws of motion, the acceleration of the *i* atom can be obtained as

(2) $$\vec{a_{i}}=\frac{\vec{F_{i}}}{m_{i}}$$

Integrating Newton’s equations of motion with respect to time enables us to obtain the velocity and the position of the *i* atom after *t* moments:

(3) $$\frac{d^{2}}{dt^{2}}\vec{r_{i}}=\frac{d}{dt}\vec{v_{i}}=\vec{a_{i}}$$

(4) $$\vec{v_{i}}=\vec{v_{i^{0}}}+\vec{a_{i}}t$$

(5) $$\vec{r_{i}}=\vec{r_{i^{0}}}+\vec{v_{i^{0}}}t+\frac{1}{2}\vec{a_{i}}t^{2}$$

$\vec{v_{i}}$ refers to the velocity of the particle, $\vec{r_{i}}$ denotes the position of the particle, and the physical quantities with the superscript “0” denote their initial values.

The above process starts by calculating the potential energy of the system, which is found by calculating the location of each molecule in the system. Calculate the force and acceleration on each particle in the system. From this, the position and velocity of the particle after time t can be obtained. Next, the above steps are repeated, and each time, the total potential energy of the system, the force, and the acceleration applied need to be calculated from the new position of the particle to obtain the new position and velocity of the particle, and so on and so forth, and the data such as the position, velocity, and acceleration of the particle movement under each time are obtained through continuous calculation to obtain the particle movement trajectory [25].

### 2.2. Simulation Techniques

#### 2.2.1. Statistical Ensemble

A statistical ensemble is a collection of a large number of independent systems, each of which is identical in nature and structure but in a different state of motion, under specific macroscopic conditions. An ensemble makes the microscopic statistical theory more universal. In MDS, macroscopic parameters such as temperature, pressure, energy, etc., are purposely set according to the research needs, and necessary constraints are imposed on the simulated system.

The canonical ensemble, abbreviated as NVT, indicates that the system has a definite number of particles (N), volume (*V*), and temperature (T) and is suitable for the study of a closed system that exchanges only heat with outside [26]. The micro-canonical ensemble, abbreviated as NVE, represents a system with a definite number of particles (N), volume (*V*), and energy (E) and is suitable for the study of closed systems without any exchange of matter or energy with outside [27]. The constant-pressure, constant-temperature ensemble, abbreviated as NPT, means that the system has a definite number of particles (N), pressure (P), and temperature (T), and the pressure is controlled by controlling the volume, which is suitable for the study of systems where the volume is constantly changing [28]. The grand canonical ensemble, abbreviated as μVT, denotes a system with a definite chemical potential (μ), volume (*V*), and temperature (T) and is suitable for the study of open systems with both mass transfer and energy exchange with outside [29].

The selection of an appropriate statistical system is crucial to ensure the accuracy and reliability of the simulation results, and different types of physical and chemical processes can be effectively simulated by setting the system conditions appropriately. As for molecular dynamics simulation on carbon dioxide applied for methane exploitation from hydrates, current challenges include ensemble selection for complex hydrate systems and computational efficiency. Future trends focus on advanced algorithms and hybrid methods to better capture system behaviors, ensuring more precise and reliable simulations.

#### 2.2.2. Molecular Force Field

In MDS, accurately solving the Newtonian mechanics equations of motion for particles first requires specifying the interactions between the particles. This setting is usually achieved through a molecular force field, which consists of a potential function model and its parameters that together define the form of the interactions between the particles. The molecular force field consists of two main parts: the bonding potential (U_bond_) and the non-bonding potential (U_non-bond_).

U = U_bonded_ + U_non-bonded_ (6)

Different parameters of the potential energy function determine the accuracy of the simulation results. A variety of molecular force fields have been developed for different types of molecules. Choosing the right force field is crucial to ensure the accuracy of the simulation results, so before starting the simulation, the appropriate force field should be selected according to the object and purpose of the study.

In MDS, choosing the right molecular force field is critical to ensure the accuracy of the simulation results. OPLS (Optimized Potential for Liquid Simulation) was proposed by Jorgensen and Tirado-Rives in 1988 for liquid systems such as peptides, proteins, nucleic acids, and organic solvents. It includes the combined atomic force field (OPLS-UA, without hydrogen atoms) and all-atom force field (OPLS-AA, with hydrogen atoms) [30]. COMPASS (Condensed-Phase Optimized Molecular Potential for Atomistic Simulation Studies) is the first force field to unify organic and inorganic molecular systems for organic small molecules, polymers, and metal compounds. It combines quantum mechanical calculations and empirical methods of liquid molecular dynamics for condensed matter simulations [31]. CHARMM (Chemistry at Harvard Macromolecular Mechanics) was created by Martin Karplus of Harvard University with parameters based on quantum mechanical calculations. It is widely used in the simulation of biological macromolecules, such as proteins, nucleic acids, and sugars [32]. AMBER (Assisted Model Building with Energy Minimization) was developed by Kollman’s group, with a simple form and a small amount of calculation. It is mainly applicable to the simulation of biomolecules such as proteins, DNA, RNA, carbohydrates, and fats but has limited application in small molecule systems [33]. CVFF (Consistent Valence Force Field) was developed by Dauber-Osguthorpe’s group at the University of Bath, UK, and is mainly used for organic small molecules and protein systems. Extensions can be used to simulate certain inorganic systems such as silicates and aluminosilicates [34].

Each molecular force field serves different research needs: OPLS excels in organic solvents and biological systems, while COMPASS is ideal for condensed-phase simulations, including some inorganic materials. CHARMM and AMBER are widely used for biomolecules, with AMBER being limited for small molecules. In contrast, CVFF supports both organic and limited inorganic systems. For carbon dioxide applied for methane exploitation from hydrates, these molecular force fields have limited suitability; they still face challenges in fully capturing the interactions between water (H_2_O), carbon dioxide (CO_2_), and methane (CH_4_), which are critical in this research. The accurate simulation of these molecules requires selecting specific molecular models tailored to their unique behaviors within hydrate environments.

SPC/E is a commonly used rigid water model proposed by Berendsen, H.J.C. et al. in 1976 [35]. The SPC/E model retains the advantages of the SPC model with modifications in charge and bond lengths to improve the ability to describe the properties of liquid water. The SPC/Fw was proposed by Ferguson, D.M. and Weare, J.H. in 1984 as an improved model [36] to better simulate the dielectric constant and surface tension of water by adjusting the charge distribution. TIP4P/Ice, proposed by Vega, C. et al. in 2009 [37], is specifically designed to simulate the intertransformation of ice and water and is suitable for hydrate studies at low temperatures. TIP4P/2005 was developed by Abascal, J.L.F. and Vega, C. in 2005 [38], aiming to improve the performance of the TIP4P model in liquid water and ice, especially under high-pressure conditions. Trappe was proposed by Harris, J.G. et al. in 2001 [39] for modeling carbon dioxide molecules and is particularly suitable for modeling carbon dioxide behavior in hydrate systems. EPM2 was developed by Stone, A.J. in 1998 [40] and is a widely used carbon dioxide model for the simulation of carbon dioxide molecules under various conditions. OPLS-AA is an all-atom force field proposed by Jorgensen, W.L. and Tirado-Rives, J. in 1988 [41], suitable for a wide range of molecular simulations including carbon dioxide. OPLS-UA combined atomic force field (OPLS-UA) was proposed by Jorgensen, W.L. and Madura, J.D. in 1988 [42] for methane molecule simulations that do not include hydrogen atoms. TraPPE-UA was developed by Chen, B. and Siepmann, J.I. in 2001 [43] and is suitable for the simulation of methane molecules under the joint atomic force field, which is widely used in hydrate studies. In hydrate modeling, SPC/E and TIP4P/2005 perform well for liquid water and ice properties under varying pressure and temperature, making them versatile choices. TIP4P/Ice is ideal for studies requiring precise ice–water transformations, essential in low-temperature hydrate simulations. Trappe and EPM2 offer robust carbon dioxide modeling; Trappe excels in hydrate systems while EPM2 adapts well to diverse conditions. For methane, OPLS-UA and TraPPE-UA are effective, with TraPPE-UA being especially prominent in hydrate research due to its all-atom accuracy. However, limitations exist, such as SPC/E’s simplified rigidity, which may impact interactions under extreme conditions.

#### 2.2.3. Numerical Solution

The core of MDS lies in solving Newton’s equations of motion numerically to track the trajectories of atoms and molecules over time. These methods approximate the equations of motion of the system and predict the dynamical behavior of the system. Mathematically, MDS is essentially an initial value problem solved using the finite difference method. During the simulation process, the positions, velocities, and accelerations of the particles are unfolded based on Taylor’s formula, which facilitates numerical calculations. Since the MDS involves the calculation of a large number of atoms, this places high demands on the time and space efficiency of the algorithm. Therefore, the selection of an appropriate numerical solution method is crucial. Such a method needs to be able to effectively handle the interactions between a large number of atoms and improve computational efficiency while ensuring computational accuracy so that accurate and reliable simulation results can be obtained within a limited time.

The Verlet algorithm is a classical explicit integration method widely used in MDS [24]. It reduces the error by alternately updating the position and velocity. The position of the atom $\vec{r}\left(t\right)$ at moment t, the position $\vec{r}\left(t-\Delta t\right)$ at moment t−Δt, and the acceleration $\vec{a}\left(t\right)$ are used to find the position at moment t+Δt. Verlet’s algorithm has good energy conservation properties, is computationally simple and stable, and is suitable for long time simulations. The choice of time step Δt in Verlet’s algorithm is relatively flexible and may be more stable for some systems.

(7) $$\vec{v}\left(t\right)=\frac{1}{2\Delta t}\left[\vec{r}\left(t+\Delta t\right)-\vec{r}\left(t-\Delta t\right)\right]$$

(8) $$\vec{r}\left(t+\Delta t\right)=-\vec{r}\left(t-\Delta t\right)+2\vec{r}\left(t\right)+\vec{a}\left(t\right){\left(\Delta t\right)}^{2}$$

The Leap-Frog algorithm is also an explicit integration method that reduces error by interleaving the updates of position and velocity. Position and velocity are updated separately in the Leap-Frog algorithm, and velocity is usually updated at “half-steps” while position is updated at “full-steps” [44,45]. The advantage of the Leap-Frog algorithm is that it is computationally efficient and can compute the trajectory of an object faster while ensuring a certain degree of accuracy. In addition, the algorithm has good stability and convergence and is suitable for the simulation of different types of physical systems, but the accuracy is slightly lower than that of Verlet’s algorithm.

(9) $$\left\{\begin{array}{c} \vec{v_{i}}\left(t+\frac{1}{2}\Delta t\right)=\vec{v_{i}}\left(t-\frac{1}{2}\Delta_{t}\right)+\vec{a_{i}}\left(t\right)\Delta t\\ \vec{r_{i}}\left(t+\Delta t\right)=\vec{r_{i}}\left(t\right)+\vec{\nu_{i}}\left(t+\frac{1}{2}\Delta t\right) \end{array}\right.$$

Synthesizing the Verlet algorithm and Leap-Frog algorithm produces the Velocity Verlet algorithm. When the position, velocity, and force of the moment t are given, the position, velocity, and force of the moment t+Δt can be calculated [26,27]. The Verlet algorithm is rewritten in such a way that position and velocity can be calculated at the same time. The Velocity Verlet algorithm is an improvement on the Verlet algorithm and improves accuracy by updating the velocity directly; the new position and force are calculated before the new velocity can be calculated.

(10) $$\vec{r}\left(t+\Delta t\right)=\vec{r}\left(t\right)+\vec{v}\left(t\right)\Delta t+\frac{f\left(t\right)}{2m}\Delta t^{2}$$

(11) $$\vec{v}\left(t+\Delta t\right)=\vec{v}\left(t\right)+\frac{f\left(t+\Delta t\right)+f\left(t\right)}{2m}\Delta t$$

Each of the three algorithms has its own advantages and disadvantages, but they all have an integration time step, so the selection of the time step is directly related to the accuracy of the simulation results and computational efficiency [29]. In addition, the principle of energy conservation is also a prerequisite for correct calculation. Therefore, the selection of a good integration algorithm needs to consider the conservation of energy, the appropriate time step, the demand for computer memory, and so on. By rationally selecting and applying these numerical solution algorithms, more accurate results can be obtained in MDS, leading to a better understanding of the kinetic behavior of molecular systems [28]. The algorithms for a numerical solution are key in molecular dynamics simulation on carbon dioxide applied for methane exploitation from hydrates, influencing accuracy and stability. The Verlet algorithm is simple but may lack precision in velocity calculations. The Leap-Frog method improves velocity accuracy but can be less stable. The Velocity Verlet algorithm balances both but can be computationally intensive. Current challenges include efficiency and scalability. Future trends focus on hybrid algorithms and parallel computing to enhance performance and accuracy.

### 2.3. Characterization Parameters

After the MDS, in addition to analyzing the conformation of the system, obtaining some characterization data of atoms or molecules in the system that can reflect the changes of the system is also an effective way to derive the corresponding mechanisms and laws. The following are some commonly used characterization parameters in MDS studies related to hydrates.

#### 2.3.1. Mean Square Displacement

Mean square displacement (MSD) quantifies how much a particle’s position changes over time relative to an idealized lattice point, making it a useful metric for evaluating particle diffusion and thermal motion in a system. MSD analysis helps track deviations from initial positions, reflecting the particle’s mobility, environmental interactions, and changes in structural order. For example, in molecular dynamics simulations, MSD provides insights into diffusion rates, phase transitions, and the stability of structures under varying temperature and pressure conditions.

(12) $$MSD\left(t\right)=\left\langle {\left(x=x_{0}\right)}^{2}\right\rangle =\frac{1}{N}\sum_{n=1}^{N}{\left(x_{n}\left(t\right)-x_{n}\left(0\right)\right)}^{2}$$

where *N* represents the number of molecules to be averaged, $x_{n}\left(0\right)$ is the reference position of each molecule, and $x_{n}\left(t\right)$ denotes the position of the molecule at the moment *t*.

The MSD curves reflect the nature of the motion of the molecules of the system during MDS, and the values of the MSDs for different molecules will be different [46]. In hydrate-related studies, since the guest molecules are trapped in the hydrate cage, their positions are relatively fixed, and their MSD values will float around zero. In studies of systems such as liquids or gases, where the molecules are not restricted in their movement, the value of MSD will increase with time.

#### 2.3.2. Radial Distribution Function

The radial distribution function (RDF) is the odds of a β-particle occurring in a spherical shell of unit thickness dr at a distance r from the α-center of any reference particle and is used to characterize the deviation of the probability density of finding another particle per unit volume around a point relative to the probability density of a uniform distribution. This in turn analyzes the average distance between particles and the strength of the interaction [27].

(13) $$g\left(r\right)=\frac{dN}{\rho 4\pi r^{2}dr}$$

where dN is the number of particles between r→dr and ρ is the density of the system.

The RDF is a parameter commonly used to analyze the structural changes of hydrates in the system; it is used to describe the orderliness of the system structure and to observe the motion of the specified particles, and it can be used to study the change of the motion of different particles in the molecular simulation process of hydrates to determine whether the hydrate is formed or decomposed [46].

#### 2.3.3. Coordination Number

The radial function is integrated to obtain the coordination number (CN) [47]. The *CN* is used to characterize the number of particles around the central particle, and the number of coordination numbers indicates the size of the distribution density of particles around the particle. The coordination number is often used as an aid in structural analysis.

(14) $$CN=4\pi \rho \int_{r_{1}}^{r_{2}}r^{2}g\left(r\right)dr$$

where $r_{1}$ and $r_{2}$ are the inner boundary and outer distance of the first coordination shell layer. Typically, $r_{1}$ can be set to zero or the starting point of the first peak, while $r_{2}$ is the first minimum point or the end point of the first peak.

*CN* is used to characterize how water molecules form a hydrated shell layer around a central gas molecule, which is essential for understanding hydrate stability and solvation mechanisms [48].

#### 2.3.4. Angular Order Parameter

The angular order parameter (AOP) is a parameter that characterizes the structure of hydrates and is also known as the three-body order parameter. The *AOP* is the parameter that measures the phase state of a system by measuring the extent to which the direction of hydrogen bond formation by water molecules deviates from the tetrahedron. The formula for calculating it is shown as follows:

(15) $$AOP=\Sigma \;[\;(|cos\theta |cos\theta )+cos^{2}\;(109.47{}^{\circ}){]}^{2}$$

where θ is the angle formed by the oxygen atom in the selected water molecule with the oxygen atoms in two other neighboring water molecules. The angular order parameter for hydrates is 0.1 [49].

In hydrates or similar structures, the *AOP* can be used to describe specific structures formed between water molecules or other solute molecules through hydrogen bonding or other interactions. These structures, which exhibit some form of cage, network, or periodic arrangement, are essential to understanding the nature and structure of hydrates. Therefore, *AOP* is often used to determine whether a substance in a system is in the hydrate state.

#### 2.3.5. Hydrogen Bonds

Water molecules in the crystal structure of a hydrate are connected to each other by hydrogen bonds, and the arrangement and combination of these bonds determine the internal structural characteristics of the hydrate. Specifically, the hydrogen bonding structure between water molecules builds water molecule cages of various shapes, which have different sizes, shapes, and arrangements and thus form different structures of hydrates, such as SⅠ, SⅡ, and SH types. In the process of hydrate generation, water molecules first form a cage structure through hydrogen bonding, and conversely, when a large amount of hydrate decomposes, the hydrogen bonding breaks, and the cage structure of hydrate is destroyed and decomposes [50]. By judging the number of hydrogen bonds formed, it is possible to determine the crystalline condition of hydrates in the system or to analyze the optimal conditions for hydrate formation or decomposition by observing the number of hydrogen bonds formed through simulations under different conditions.

## 3. Mechanism of Carbon Dioxide Replacement for Hydrate Exploitation

In the study of carbon dioxide replacement for hydrate exploitation, two main mechanisms have been identified: the guest molecular replacement mechanism [51] and the hydrate reconfiguration mechanism [52]. These mechanisms highlight different aspects of molecular exchange during the replacement process and serve as a theoretical foundation for optimizing carbon dioxide replacement technology in hydrate exploitation. MDS has proven advantageous offering high-resolution observation, microscopic mechanism analysis, dynamic process visualization, and structural evolution tracking. Through these capabilities, researchers gain deeper insights into the molecular motions involved in the replacement process, verify experimental findings, and elucidate the mechanisms underlying structural changes in hydrates. Consequently, significant advancements have been achieved in understanding and applying MDS to hydrate studies.

### 3.1. Guest Molecular Replacement

**Figure 3:**
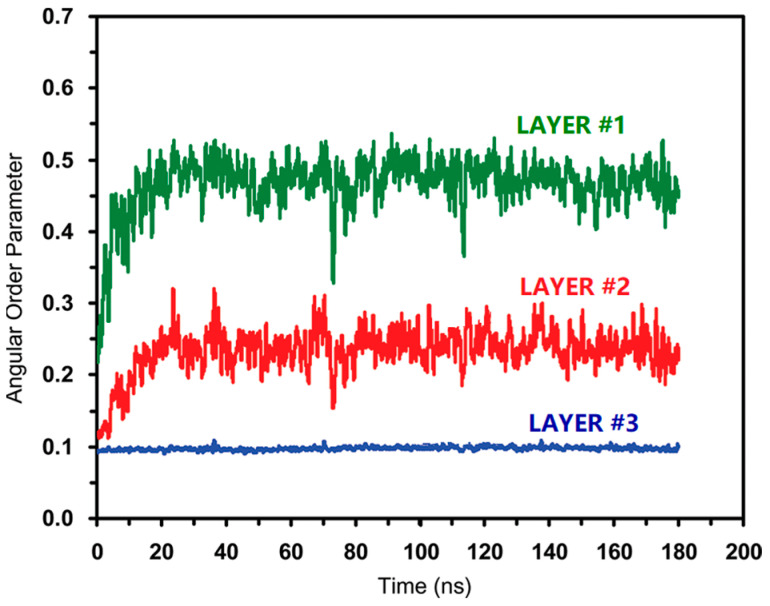
Water molecule angular order parameters for different hydrate layers (Tung, Y.T. et al., 2011 [54]).

MDS enables high-resolution observations of dynamic changes at the molecular level allowing researchers to visualize how carbon dioxide molecules penetrate the hydrate cage while displacing methane. MDS elucidates microscopic mechanisms, such as the interaction forces between carbon dioxide and the hydrate cage walls, facilitating methane release. Additionally, MDS provides dynamic visualizations of the substitution process, enhancing understanding of molecular motions and validating experimental observations [53]. Tung, Y.T. et al. [54] performed a long time (180 ns) simulation of carbon dioxide substitution of natural gas hydrate using LAMMPS software V.17Jan2005 at a constant pressure of 6 MPa and heating from 200 to 280 K at a rate of 0.5 K/ps in the NPT system synthesis. The angular order parameters of water molecules in the hydrate cage during carbon dioxide substitution were investigated (Figure 3).

The AOP of water in liquid phase, hydrate, and ice forms are 0.8, 0.1, and 0. The results show that the hydrogen bonding framework is not decomposed during the substitution process, and at the surface of the hydrate layer (Layer #1/Layer #2), the AOP parameter of the cage structure of the hydrate is larger, the water molecules are more mobile, and the hydrate cage is softer, but compared to liquid water (AOP 0.8), there is still a considerable gap in the hydrate surface layer carbon dioxide, and methane molecules are directly replaced. In contrast, the cage structure of hydrate away from the surface of the hydrate layer (Layer #3) is rigid, and the replacement of carbon dioxide and methane is accomplished by transient co-occupation within the cage. The water molecules in the surface layer (Layer #1/Layer #2) have high AOP values, so the instability of the hydrate cage in the surface layer can be seen. However, the AOP of the water molecules in the surface layer is significantly lower than the AOP of liquid water (0.8), which indicates that there is no fully dissolved liquid water at the phase interface. The AOP of water molecules in Layer #3 is the same as that of crystalline hydrates (0.1), suggesting a stable hydrate cage in the inner layer.

Zhang, K. et al. [55] conducted an MDS study of carbon dioxide replacement of methane hydrate to investigate how this replacement process occurs under different temperature conditions (Table 1). It was found that the number of water molecules in the hydrate remains relatively stable at low temperatures of 250 K when carbon dioxide molecules enter the hydrate structure to replace the methane molecules. This means that the hydrate cage does not dissociate during this process, but rather a direct exchange of guest molecules (i.e., carbon dioxide replacing methane) occurs. In addition, the number of water molecules in the hydrate decreased slightly when the temperature increased, suggesting that some dissociation of the hydrate cage structure may occur as the temperature increases. This finding is consistent with previous findings by Tung et al. [54] and further supports the idea that gas molecule replacement can be achieved without complete dissociation of the hydrate cage.

**Figure 4:**
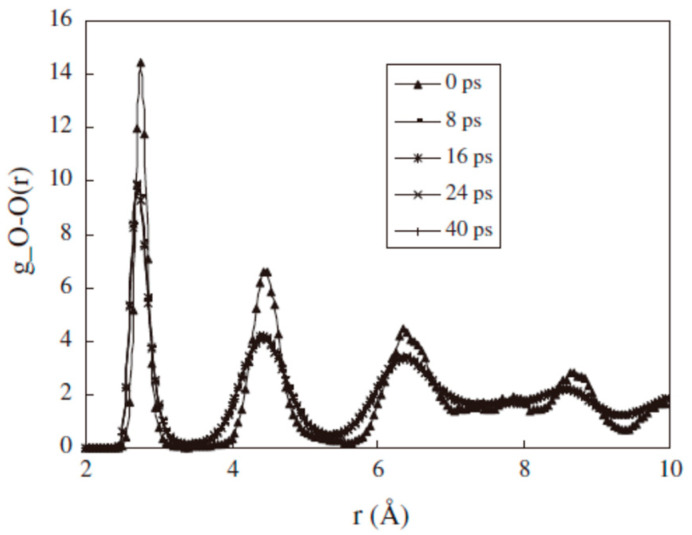
Variation in the oxygen atom pair distribution function during simulation (Qi, Y. et al., 2011 [56]).

Qi, Y. et al. [56] used MDS to quantify the process of carbon dioxide replacement of natural gas hydrates. Their study focused on the distribution function of oxygen atom pairs as a function of time. From the results of the simulations, the shape of the curve of the distribution function of oxygen atom pairs remained relatively consistent throughout the time range of 0 to 40 ps, except for the variation in the peak height at the initial moment (0 ps). This means that the thermal movement of water molecules at the substitution interface during the initial period of substitution resulted in a decrease in the peak height, but this did not affect the shape of the overall distribution function. From this observation, it can be concluded that the basic structure of the hydrate is not significantly altered or decomposed during carbon dioxide replacement of methane. Instead, guest molecules within the hydrate (e.g., methane) are replaced by carbon dioxide molecules, while the hydrate cage structure retains its integrity. This finding emphasizes that the replacement process can be accomplished by direct exchange between molecules, rather than by disrupting the hydrate structure (Figure 4).

**Figure 5:**
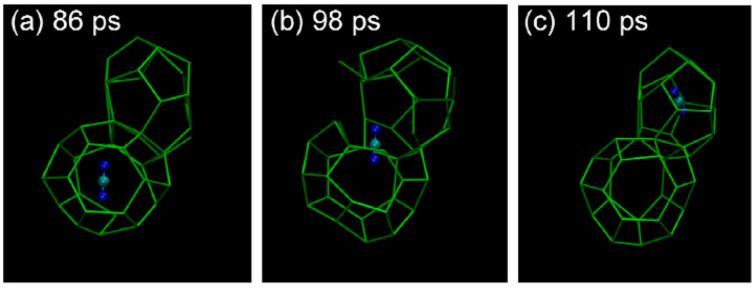
Trajectories of carbon dioxide molecules in hydrate cages at 315 K (Liang, S. et al., 2016 [57]. Where green wireframe represents cage structure of hydrate, combination of one green ball and two blue balls represents carbon dioxide molecule).

Liang, S. et al. [57] investigated the transport mechanism of carbon dioxide in hydrates by combining MDS and transition state theory. Their study showed that carbon dioxide molecules were able to transport between hydrate cages under different temperature conditions and that the entry of carbon dioxide molecules into the hydrate cages did not cause significant changes in the hydrate cage structure in the process. Specifically, the trajectories of carbon dioxide molecules at a temperature of 315 K show that these molecules are able to move smoothly between hydrate cages without causing dramatic changes in the cage structure (Figure 5). This suggests that carbon dioxide molecules can effectively penetrate into the hydrate structure and undergo replacement reactions with other molecules (e.g., methane), while maintaining the overall structural stability of the hydrate.

**Figure 6:**
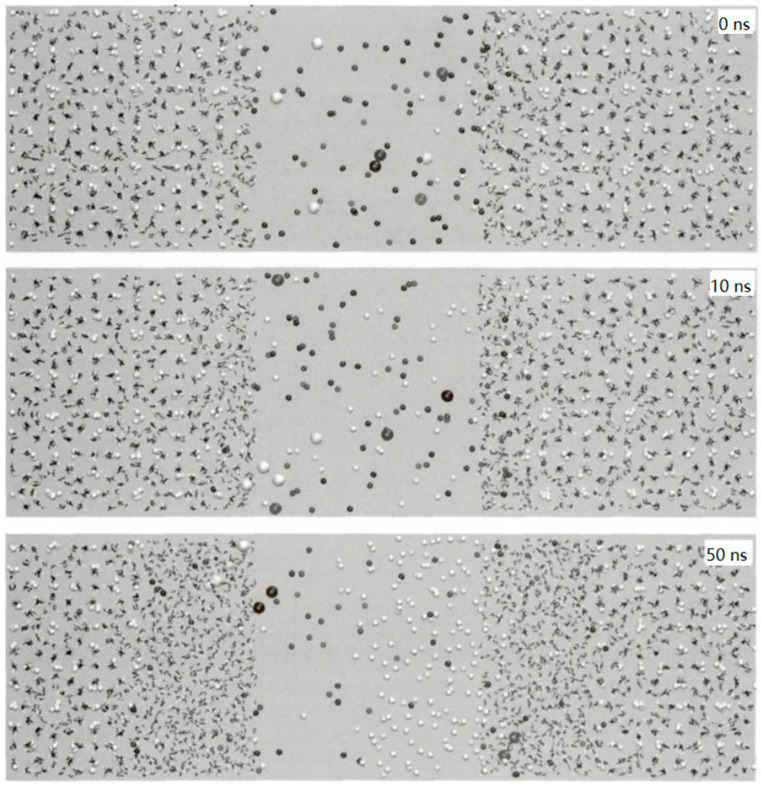
Conformational diagram of the system for the replacement of methane hydrate by carbon dioxide in NaCl solution (Yi, L. et al., 2016 [58]).

Using a molecular dynamics approach and through LAMMPS software, Yi, L. et al. [58] investigated the process of carbon dioxide replacement of methane hydrate in a NaCl solution environment. Their results show that in the presence of sodium (Na^+^) and chloride (Cl^−^) ions, the methane hydrate cage at the hydrate phase interface undergoes a certain amount of deformation to form cracks (Figure 6). This deformation allows carbon dioxide molecules to enter the hydrate cage directly and displace the methane molecules that would otherwise occupy the cage sites.

This finding suggests that in the presence of salt solutions (e.g., NaCl solution), the action of ions may promote localized deformation of hydrate cages, which facilitates the insertion of carbon dioxide and the release of methane. The understanding of this mechanism has important implications for the optimization of natural gas hydrate extraction strategies as well as carbon dioxide sequestration technologies. In this way, effective replacement of gas molecules can be achieved without completely destroying the hydrate structure.

### 3.2. Hydrate Reconfiguration

**Figure 7:**
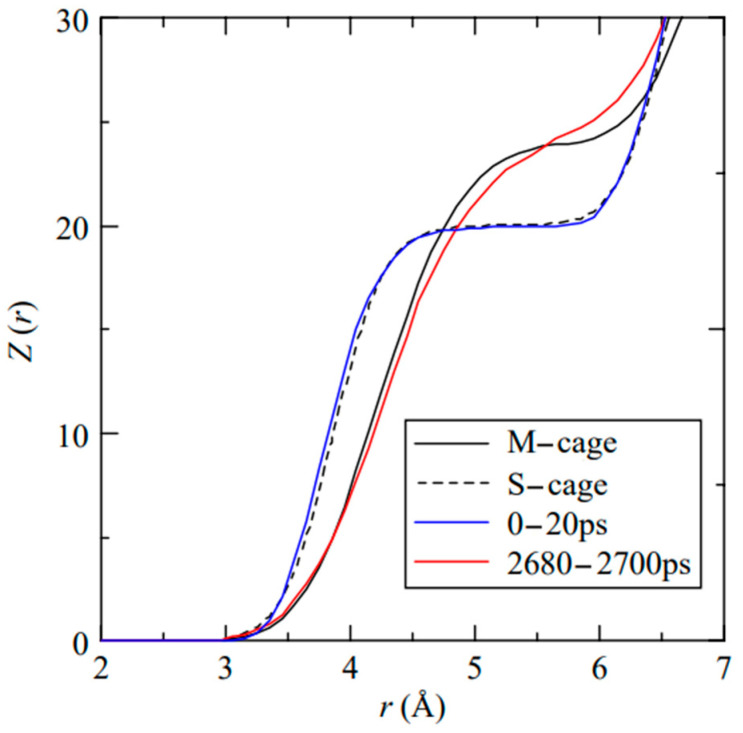
Coordination number of water molecules in hydrates (Iwai, Y. et al., 2012 [59]).

Iwai, Y. et al. [59] investigated the coordination number of water molecules in hydrates during carbon dioxide substitution of SⅠ methane hydrate by MDS under NPT system conditions. The temperature and pressure conditions set for the study were 270 K and 5.0 MPa, and the molecular numbers of water, methane, and carbon dioxide were 736, 128, and 192, respectively. The results of the study showed that during the replacement of methane by carbon dioxide, the melting phenomenon occurred in the cage structure of the hydrate, and a liquid water phase was produced (Figure 7).

At the junction of the liquid water phase and the hydrate phase, the collapse of small cages in the SⅠ type hydrate was observed after the carbon dioxide molecules entered and displaced the methane molecules therein. Subsequently, these small cages transformed into large cages, changing the overall structure of the hydrate cage.

This finding supports a different view that the cage structure of hydrates is not simply maintained by the direct replacement of guest molecules during carbon dioxide replacement of methane hydrates, but may be completely destroyed with the concomitant production of free water molecules.

**Figure 8:**
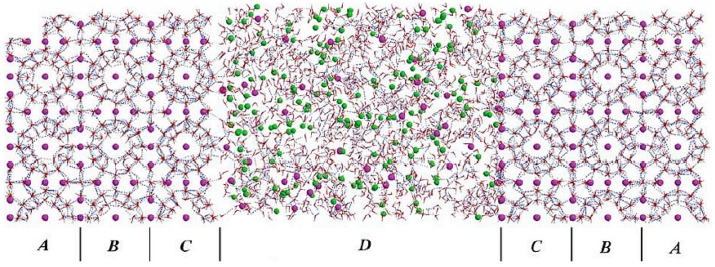
Initial system conformation (Bai, D. et al., 2012 [60]).

Bai, D. et al. [60] carried out MDS using LAMMPS software in the NPT system at 270 K and 20 bar to analyze how carbon dioxide interacts with methane hydrate and gradually displaces the methane molecules in it, in order to investigate the mechanism of carbon dioxide displacement of methane hydrate. According to their study, Figure 8 shows the initial conformation at the beginning of the simulation, in which the D region represents the liquid phase and the ABC region is the methane hydrate phase in this system.

**Figure 9:**
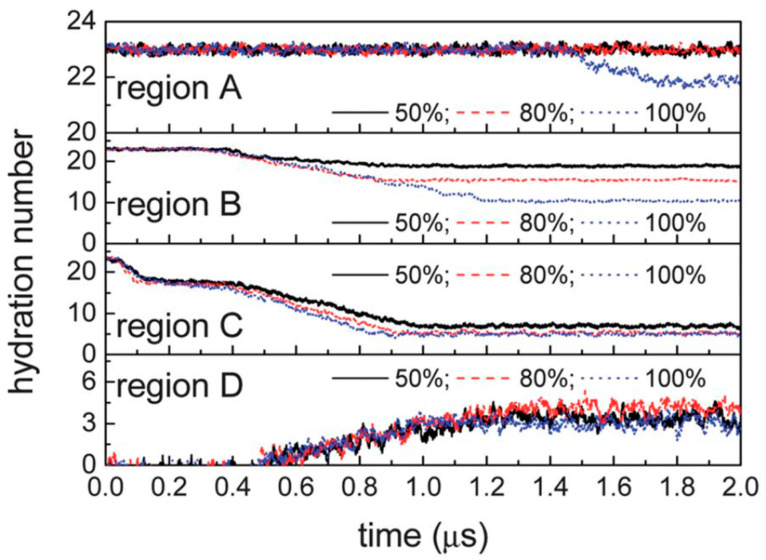
Hydration numbers in different regions (Bai, D. et al., 2012 [60]).

Figure 9 illustrates the evolution of the hydration number of methane molecules in different regions over time. The hydration number here is defined as the average number of water molecules within a radius of 4.33 Å from a guest molecule. For the SI methane hydrate, the average hydration number is about 23 (24 for the six large cages and 20 for the two small cages). The hydration number in regions C and B decreases significantly with time as the replacement process proceeds (Figure 9). This implies that the hydrate structures within these two regions dissolved during the carbon dioxide replacement of methane. This observation supports the idea that the hydrate cage structure changes during the replacement of methane hydrate by carbon dioxide and suggests that the original hydrate structure gradually disintegrates as the replacement occurs, leading to a decrease in the hydration number.

**Figure 10:**
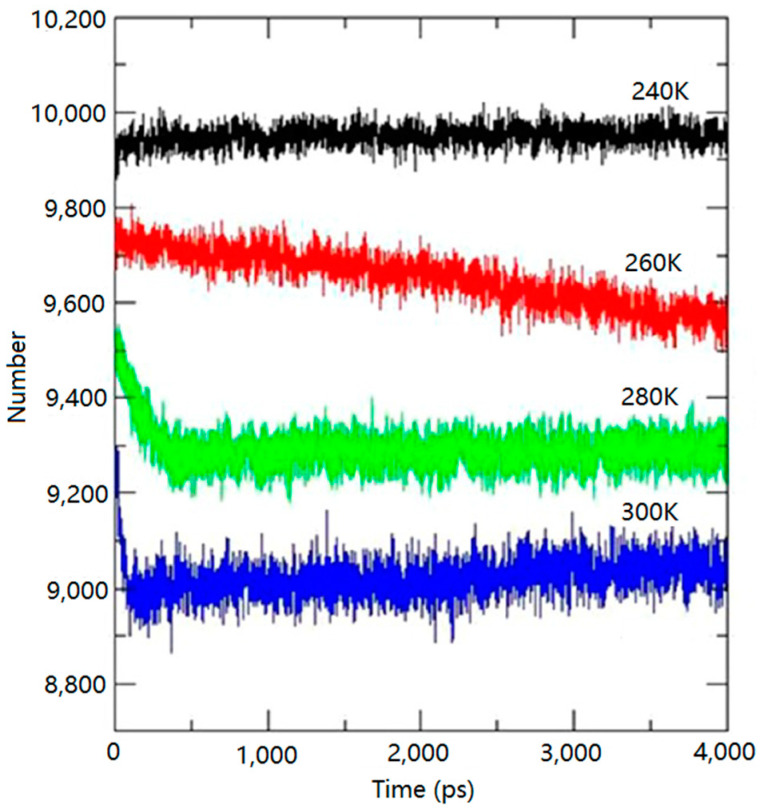
Variation in the number of hydrogen bonds in methane hydrates with simulation time under different temperature conditions (Uddin, M. et al., 2014 [61]).

Uddin, M. [61], in his MDS study on the dissociation of gas hydrates, found that the higher the temperature, the faster the rate of cleavage of the hydrogen bonding network of the hydrate in the system under the same pressure conditions. This finding can be visualized in Figure 10, which shows that the rate of hydrogen bonding network cleavage accelerates with increasing temperature, implying a significant effect of temperature on hydrate stability. Higher temperatures accelerate the breaking of hydrogen bonds within the hydrate structure, thus facilitating the hydrate decomposition process. This finding is important for understanding and controlling the behavior of hydrates under different environmental conditions.

**Figure 11:**
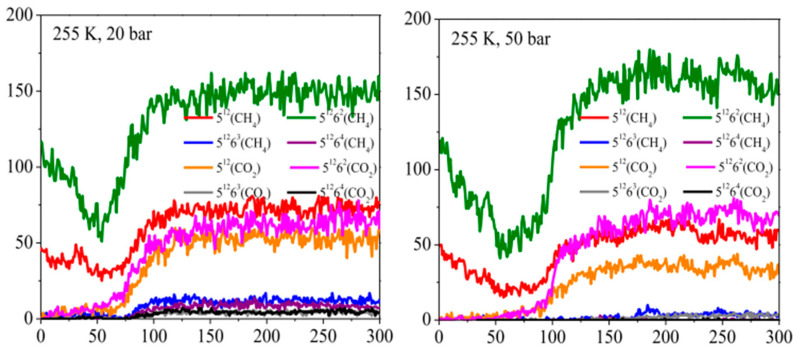
Variation in the number of hydrate cages for pressures of 20 bar and 50 bar at 255 K temperature (Wu, G. et al., 2019 [62]).

The study by Wu, G. et al. [62] simulated the process of carbon dioxide replacement of methane hydrate by molecular dynamics at six different temperatures (250–275 K) and different pressures (20, 50, and 100 bar). During the simulations, they observed a brief dissociation of methane hydrate. Due to the very short dissociation time, the methane molecules released by dissociation quickly rejoined the hydrate cage, forming a methane–carbon dioxide mixed hydrate (Figure 11). Within the first 50 ns after the start of the simulation, the amount of methane hydrate in the cage decreased rapidly. However, the amount of carbon dioxide hydrate in the cage did not rise to a level equal to the decline in the amount of methane hydrate during the same time period. This suggests that the formation of carbon dioxide hydrate occurred after the dissociation of methane hydrate. In addition, the methane hydrate quantity rose rapidly after a short decline, forming a ‘co-growth’ phenomenon with the carbon dioxide hydrate. This finding reveals a dynamic relationship between methane hydrate dissociation and carbon dioxide hydrate formation during carbon dioxide replacement of methane hydrate. Specifically, methane hydrate undergoes a dissociation phase, followed by carbon dioxide entering the vacated cage to form a new hydrate, while methane may re-enter the neighboring cage, leading to a rebound in the amount of methane hydrate.

In summary, there are no agreed-upon conclusions on the specific mechanism of carbon dioxide replacement of methane hydrates. However, on the basis of available MDS studies, it can be tentatively surmised that the realization of the carbon dioxide substitution mechanism may depend on whether the thermodynamic conditions required for substitution are achieved. Under lower temperature conditions, hydrogen bonds do not have sufficient energy to dissociate. In this case, the contact of carbon dioxide with methane hydrate leads to a distortion or partial disruption of the cage structure of methane hydrate, mainly through intermolecular van der Waals forces. This process allows carbon dioxide to enter the hydrate cage directly and displace methane. After the replacement is complete, the hydrate cage structure remains relatively stable, and no free water molecules are produced. At higher temperatures, the hydrogen bonds have enough energy to continue to dissociate. At this point, the hydrate cage structure melts, forming a liquid water phase. During this process, the generated free water molecules combine with carbon dioxide to form a new hydrate structure. This process is usually accompanied by large structural changes, which may lead to the disintegration of the original hydrate structure and the formation of a new hydrate phase. These preliminary speculations provide a direction for further research, and future studies can continue to delve deeper into the specific replacement mechanisms under different temperature and pressure conditions and verify these hypotheses through a combination of experiments and simulations. Through such studies, the mechanism of carbon dioxide replacement of methane hydrates can be better understood and provide theoretical guidance for practical applications.

## 4. Key Issues for Carbon Dioxide Applied for Methane Exploitation from Hydrates

One of the key issues in the application of carbon dioxide for methane exploitation from hydrates is the effect of thermodynamic conditions, auxiliary gases, and other factors on the rate of carbon dioxide replacement of methane. MDS shows a unique advantage in this regard, providing detailed information at the atomic level and revealing the microscopic mechanisms involved in the substitution process. Similarly, studies by Wang, Z. et al. [63] and Pessôa Filho, P.A. et al. [64] utilize molecular-level insights to elucidate complex system behaviors, demonstrating the versatility and power of molecular dynamics simulations in diverse fields. The consistency and reliability of the results can be verified by repeating the simulation under controlled conditions. In addition, MDS can cover a wide range of conditions and scenarios, providing a solid theoretical basis for experimental design. Compared with traditional experimental tests that require a lot of time and resources, MDS is able to obtain a large amount of data in a relatively short period of time. These studies not only deepen our understanding of the process of carbon dioxide replacement in methane hydrates and its mechanism, but also provide valuable support for the optimization of technologies of carbon dioxide applied for methane exploitation from hydrates.

### 4.1. Thermodynamic Conditions

The replacement of methane hydrate by carbon dioxide is a complex multiphase, multicomponent chemical process. In this process, several factors jointly influence the replacement efficiency. Among them, thermodynamic conditions, especially temperature and pressure, are key factors affecting the replacement efficiency. Temperature has a significant effect on hydrate stability and the diffusion of gas molecules through the hydrate. Higher temperatures reduce the stability of the hydrate and may lead to its decomposition; conversely, lower temperatures help to stabilize the hydrate but may slow down the rate of diffusion of gas molecules. Pressure is also critical to the presence of hydrates and the replacement process. Higher pressures favor the formation of hydrates and the diffusion of carbon dioxide into the hydrate cages; however, if the pressure is too high, it may lead to rupture of the hydrate cages or structural instability. In addition, the combined effect of temperature and pressure determines the thermodynamic stability window of hydrates. Under specific temperature and pressure conditions, hydrates can be stable; when conditions deviate from this range, hydrates may decompose, affecting the replacement efficiency. Therefore, precise control of temperature and pressure is important in the design and implementation of carbon dioxide replacement of methane hydrates.

**Figure 12:**
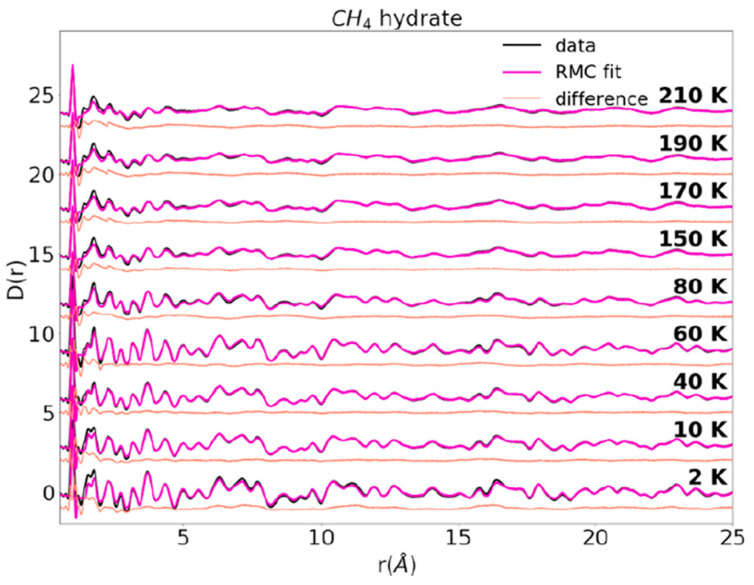
RDF values for methane hydrate at 2–210 K (Cladek, B.R. et al., 2021 [65]).

Cladek, B.R. et al. [65] used MDS to analyze the effect of guest molecules on the local structure in methane hydrates, carbon dioxide hydrates, and methane–carbon dioxide mixed hydrates in the temperature range of 2–210 K. They found that the effect of guest molecules on the local structure is very strong in methane–carbon dioxide mixed hydrates. As shown in Figure 12, when analyzing the RDF data of these three hydrates, they found that the methane–carbon dioxide mixed hydrate has the highest degree of disorder at low temperatures, which gradually diminishes with increasing temperature. This suggests that at low temperatures, the structure of the mixed hydrate is looser and becomes more ordered as the temperature increases, and that the presence of carbon dioxide increases the overall stability of the hydrate. At low temperatures, the presence of carbon dioxide makes it difficult to detach the remaining methane in the small cage from the hydrate structure, resulting in low methane recovery. The carbon dioxide hydrates and methane–carbon dioxide mixed hydrates formed during the replacement process not only contain residual methane in their own structures, but also form a barrier to the subsequent replacement of unreacted carbon dioxide and methane hydrates. This means that during the replacement process, the new hydrate structure may hinder further replacement reactions, thus affecting the overall replacement efficiency and methane recovery.

In order to achieve quantification of the replacement efficiency of carbon dioxide for methane hydrate, Zhang, K. et al. [55] performed MDS at three different temperatures, where they calculated the number of hydrate cages occupied by methane and carbon dioxide by identifying whether the methane or carbon dioxide molecules contained 10 or more hydrate water molecules within a radius of 5.5 Å. The results of the MDS are presented in Table 2. The results of the study show that the rate of substitution reactions between carbon dioxide and methane is much faster at 270 K than at 250 K. Before equilibrium is reached, the replacement reaction rate at 270 K is about 1.5 times higher than that at 250 K. These results suggest that the faster rate of the replacement reaction at higher temperatures (e.g., 270 K) may be due to the fact that higher temperatures provide more energy, which makes it easier to break the hydrogen bonds, thus facilitating the diffusion and replacement of the gas molecules. At lower temperatures (e.g., 250 K), the rate of substitution is slower, which may be due to the higher stability of hydrogen bonds, which require more energy to complete the substitution process.

**Figure 13:**
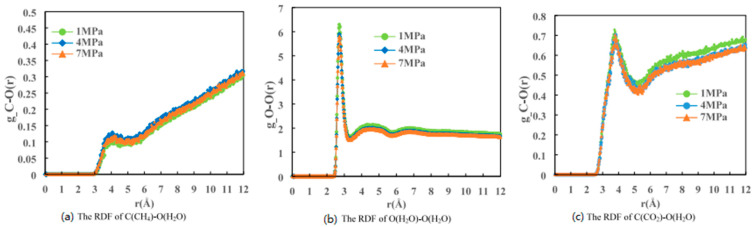
RDF of particles in the system at 3MPa with different temperatures (Guo, P. et al., 2022 [66]).

Guo, P. et al. [66] carried out MDS of carbon dioxide substitution for natural gas hydrate by Material Studio 2019 software and investigated the effects of three temperatures, 270 K, 280 K, and 290 K, on the substitution reaction at 3 MPa pressure using RDF and mean square displacement. The first characteristic peak of the RDF of methane hydrate at different temperatures is around r = 4.21 Å. The RDF of methane hydrate at different temperatures is around r = 4.21 Å. The peak value of the first peak of the RDF gradually decreases with increasing temperature, indicating that the higher the temperature, the more unstable methane hydrate is and the easier it decomposes (Figure 13). Meanwhile, the RDF of carbon dioxide hydrate gradually decreases with increasing temperature, indicating that the stability of carbon dioxide hydrate decreases at high temperatures. Therefore, among these three temperatures, the optimum temperature for the formation of carbon dioxide hydrate is 270 K. The mean square displacement curves show that the methane hydrate is in an unstable state and decomposes gradually with the increase in temperature at a pressure of 3 MPa. The mobility of methane, carbon dioxide, and water molecules increases with increasing temperature, but the increase in mobility of water molecules is relatively small, and the increase in mean square displacement of water molecules is relatively small, which is consistent with the results presented by the RDF values of water molecules, indicating that the temperature damage to the hydrate cage is relatively small.

**Figure 14:**
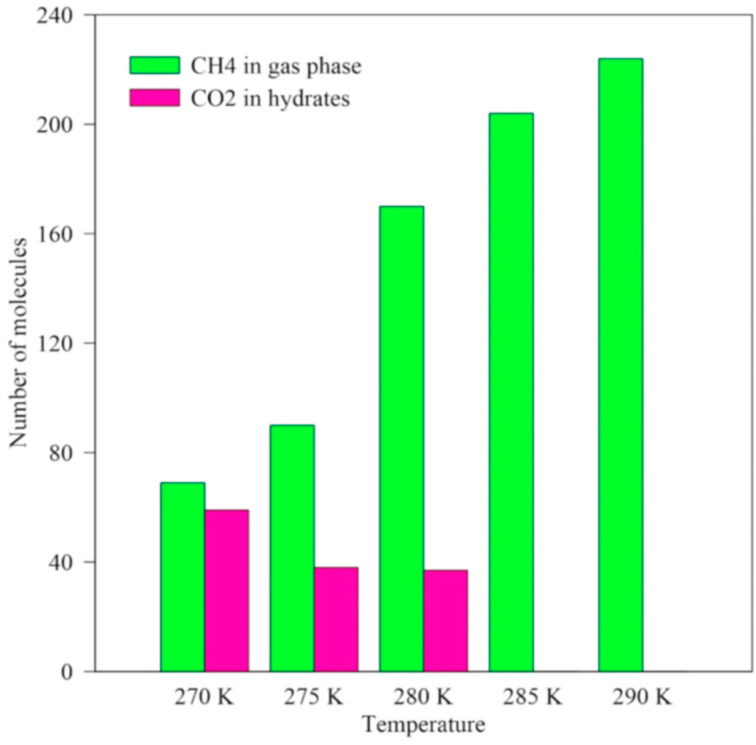
Changes in the number of methane and carbon dioxide molecules with temperature (Gajanayake, S. et al., 2022 [67]).

Gajanayake, S. et al. [67] performed MDS using the GROMACS platform to investigate the effect of different temperatures on the process of carbon dioxide replacement in natural gas hydrates. As shown in Figure 14, at 270 K, 59 carbon dioxide molecules were stored in the hydrate, and 69 methane molecules were released into the free gas phase. The number of methane molecules released into the gas phase increases with increasing temperature. However, the number of methane molecules increased significantly at 285 K and 290 K. This is due to the instability of the hydrate at higher temperatures resulting in the decomposition of a large number of hydrates, while the number of carbon dioxide molecules stored in the hydrate structure also decreased with increasing temperature. At lower temperatures, the structural stability of the hydrate is higher due to the lower kinetic energy of the gas molecules.

The sequestration of carbon dioxide molecules is higher at 270 K compared to other temperature conditions. From the point of view of methane recovery, 280 K is the optimum replacement reaction temperature because, at this temperature, a large amount of methane hydrate undergoes thermal dissociation, resulting in the release of methane into the gas phase. However, from the viewpoint of carbon dioxide sequestration, 270 K is the optimal temperature because, at this temperature, the replacement ratio is close to 1.0, which enables stable sequestration of carbon dioxide in the hydrate.

**Figure 15:**
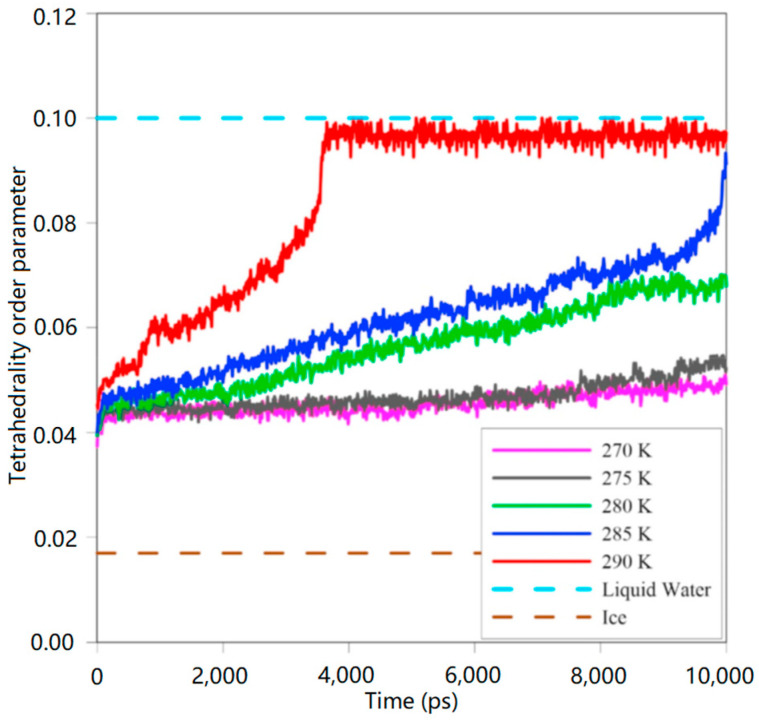
Variation in tetrahedrality order parameter with temperature at 8 MPa pressure (Gajanayake, S. et al., 2022 [67]).

In addition, the tetrahedrality order parameter (TOP) was analyzed at different temperature conditions at 8 MPa (Figure 15). It was found that when the temperature was increased above 285 K, the hydrate phase dissolved into a liquid phase, which was unfavorable to the formation of carbon dioxide hydrate. Therefore, 270 K is the optimal temperature for the replacement reaction, taking into account the methane recovery and carbon dioxide sequestration efficiency.

**Figure 16:**
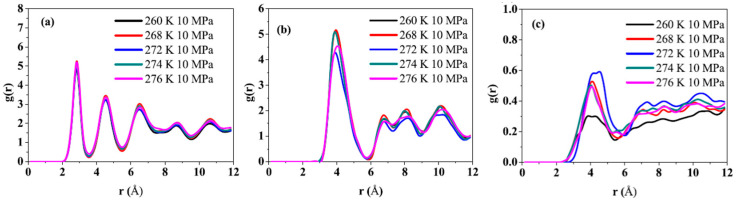
RDF of hydrate oxygen atom pairs (**a**), methane hydrate (**b**), and carbon dioxide hydrate (**c**) at different temperatures (Cheng, L. et al., 2024 [68]).

Cheng, L. et al. [68] performed molecular simulations using LAMMPS to investigate the carbon dioxide substitution process of methane hydrate at different temperatures above the methane hydrate phase equilibrium line. As shown in Figure 16, they investigated the RDFs of hydrate oxygen atom pairs, methane hydrate, and carbon dioxide hydrate under the conditions of 260 K, 268 K, 272 K, 274 K, and 276 K at a pressure of 10 MPa. It can be seen from the figure that at 272 K, the peak of methane hydrate is significantly lower than the other temperatures, while the peak of carbon dioxide hydrate is significantly higher than the other temperatures. This indicates that methane hydrate is the least stable at 272 K, while carbon dioxide hydrate is the most stable. Therefore, 272 K is the optimum temperature for methane hydrate decomposition and carbon dioxide hydrate formation compared to other temperatures in the experiment.

### 4.2. Auxiliary Gases

**Figure 17:**
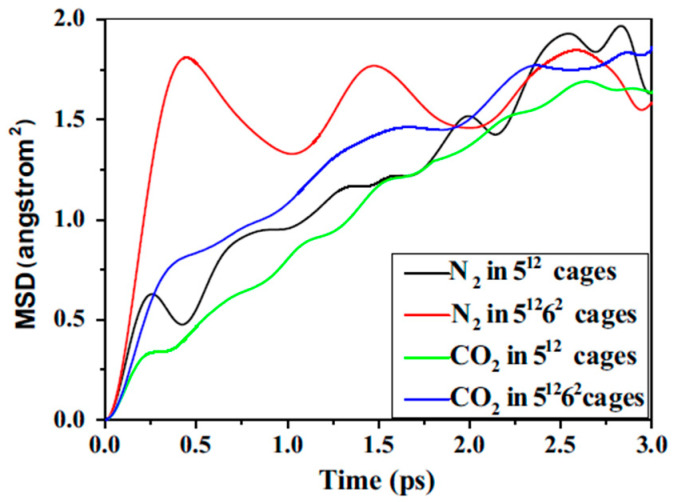
MSD of nitrogen and carbon dioxide in nitrogen hydrate and carbon dioxide hydrate (Liu, J. et al., 2016 [70]).

The main reason for the inefficiency of methane hydrate replacement is that methane molecules are more stable in small cages and are difficult to replace by other gas molecules. When attempts are made to forcibly crowd these cages with carbon dioxide, this leads to the deformation of the hydrate cage structure, which in turn prevents the entire hydrate from remaining stable. This structural instability is the key to the inefficiency of the replacement. In order to overcome this challenge, researchers proposed a method of adding other gases to the replacement gas. Experiments have shown that adding nitrogen (N_2_) is an effective strategy. Nitrogen has a tendency to occupy small cages in methane hydrates during the replacement process, and the hydrate structure can still remain stable after replacement. This finding provides a new idea to improve the replacement efficiency [16]. Further studies, such as thermodynamic and kinetic analyses using MDS by Liu, J. et al. [69], revealed the feasibility of methane hydrate replacement by nitrogen–carbon dioxide gas mixtures. They found that the Gibbs free energy of methane replacement using nitrogen–carbon dioxide gas mixture in small and large cages was negative, which proved the feasibility of the process from the thermodynamic point of view. Meanwhile, the diffusive motion of nitrogen in hydrates is faster than that of carbon dioxide, which implies that nitrogen acts as a synergistic replacement kinetically, as demonstrated by the differences in the MSD (Mean Square Displacement) of nitrogen and carbon dioxide in nitrogen hydrate and carbon dioxide hydrate, shown in Figure 17 [70]. Therefore, the replacement of methane hydrate by a nitrogen–carbon dioxide gas mixture is thermodynamically dominated by carbon dioxide replacement, while kinetically synergistic replacement is performed by nitrogen.

The study by Matsui, H. et al. [71] provides further insight into the effect of nitrogen gas on the carbon dioxide displacement process of methane under specific conditions (280 K and 6 MPa), which provides important information for our understanding of the gas mixture displacement mechanism as well as optimization strategies in practical applications. Firstly, they found that methane was recovered more efficiently when using gas mixtures for substitution compared to pure carbon dioxide gas. This finding validates the advantages of gas mixtures in improving the replacement efficiency. The nitrogen in the gas mixture helps to facilitate the release of methane molecules due to its faster diffusion rate and tendency to occupy small cages, thus increasing the recovery rate. However, the study also pointed out the possible problems associated with excessive addition of nitrogen. When too much nitrogen is added, it can lead to the collapse of the hydrate structure. This is because the excess nitrogen may destabilize the hydrate cage structure so that it cannot continue to maintain its original form. Once the hydrate structure collapses, not only will it not be able to effectively sequester carbon dioxide, but it may also affect the entire replacement process. On the other hand, if the amount of nitrogen added is too small, the synergistic replacement effect cannot be fully utilized; thus, the methane recovery rate cannot be effectively improved. Therefore, in practical applications, it is necessary to find a suitable balance point of nitrogen addition to ensure that the methane recovery rate can be improved while the stability of the hydrate structure can be maintained to achieve effective carbon dioxide sequestration. The study by Matsui et al. [71] highlights the need to consider both methane recovery and carbon dioxide sequestration effects when adding nitrogen as a replacement gas in practical mining operations. By precisely controlling the amount of nitrogen, it is possible to ensure the replacement efficiency while avoiding the destruction of the hydrate structure and the reduction in the carbon dioxide sequestration effect.

**Figure 18:**
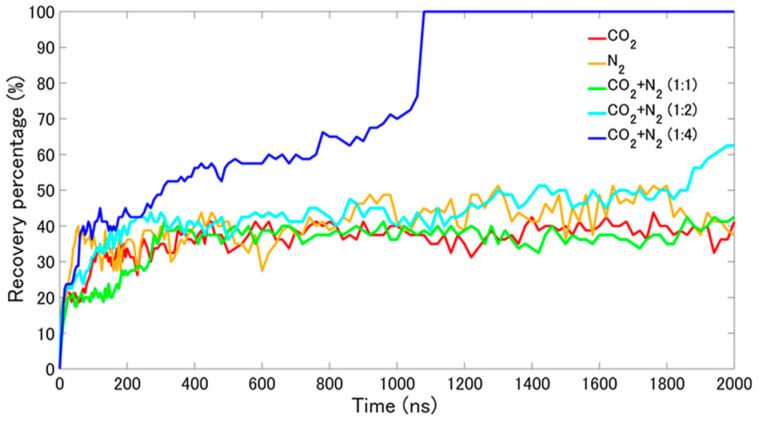
Recovery of methane in the replacement of natural gas hydrates with pure nitrogen, pure carbon dioxide, and nitrogen–carbon dioxide gas mixtures in different ratios (Matsui, H. et al., 2016 [71]).

When the ratio of nitrogen to carbon dioxide in the gas mixture reaches 1:4, the structure of the methane hydrate completely collapses after the simulation time reaches 1000 ns, and the methane gas is completely released, and the replacement efficiency reaches 100% as shown in Figure 18 [71]. However, this result also brings a problem: after the complete decomposition of methane hydrate, carbon dioxide hydrate cannot be formed again. From the point of view of carbon dioxide gas sequestration, this result is not applicable to practical development and application. The simulation results show that the nitrogen–carbon dioxide gas mixture is able to replace methane in methane hydrate very efficiently under the specific gas mixture ratio and simulation conditions and achieves a very high replacement efficiency. However, this high replacement efficiency comes at the cost of complete destruction of the methane hydrate structure and the inability to convert it to carbon dioxide hydrate.

**Figure 19:**
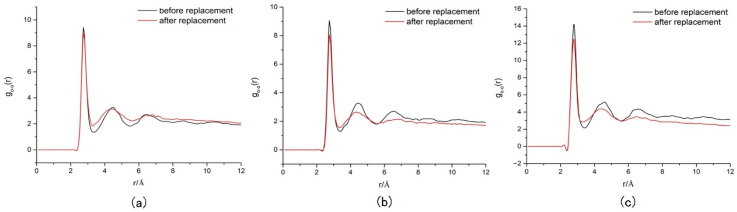
RDF of pure carbon dioxide (**a**), pure nitrogen (**b**), and nitrogen–carbon dioxide gas mixtures (**c**) before and after gas hydrate replacement (Song, W. et al., 2020 [72]).

The study by Song, W. et al. [72] enables a better comprehension of the dynamic process of gas hydrate replacement by nitrogen–carbon dioxide gas mixtures in shallow marine permafrost, especially the results of MDS at 270 K and 2 MPa. These findings are important for understanding the gas mixture replacement mechanism and optimizing the replacement process. Firstly, it was noted that the diffusion rate of carbon dioxide significantly doubled when a nitrogen–carbon dioxide gas mixture was used for the replacement compared to pure carbon dioxide gas. This finding explains why the gas mixture is more effective in displacing methane from natural gas hydrates. The increased carbon dioxide diffusion rate means that more carbon dioxide molecules are able to enter the hydrate cage faster and undergo replacement reactions with methane molecules. Secondly, the study reveals the change in hydrate stability during the replacement process through the analysis of the RDF, where the main peak of the RDF becomes lower and wider with time, which indicates that the stability of the hydrate structure decreases after the replacement. This decrease in stability may be due to the introduction of the gas mixture leading to local perturbation or reconstruction of the hydrate cage structure. However, it is worth noting that, despite the decrease in the overall stability of the hydrate after substitution, the first peak value of the RDF of water molecules after substitution of the nitrogen–carbon dioxide gas mixture is the largest (12.31), being higher compared to both the pure carbon dioxide (8.98) and pure nitrogen (8.05) substitution. This finding suggests that nitrogen may have played some stabilizing role during the gas mixture substitution process, making the substituted hydrate structure more stable in local regions instead. This may be due to the stronger interaction force between nitrogen molecules and water molecules, or the nitrogen molecules occupying some specific positions during the substitution process, which enhances the stability of the hydrate structure (Figure 19).

**Figure 20:**
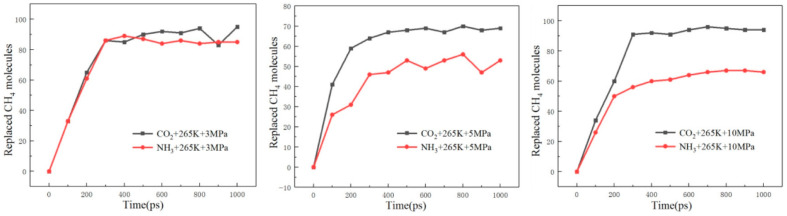
Number of replaced methane molecules varies with time, 265 K (Li, D. et al., 2021 [73]).

Li, D. et al. [73] systematically compared the efficiency of carbon dioxide and ammonia in the replacement of natural gas hydrates at different temperatures and pressures through MDS. It was found that ammonia outperforms carbon dioxide in terms of the number of methane molecules displaced and the displacement rate at a lower temperature (245 K). Since the interaction of ammonia molecules with the hydrate cage structure is stronger at low temperatures, it makes it easier for ammonia molecules to enter and occupy the methane molecules. As the temperature increases (up to 255 K), the ammonia replacement rate shows a trend of lower and then higher, but the number of methane molecules finally replaced is still higher than that of carbon dioxide. This suggests that changes in temperature have different effects on the replacement efficiency of ammonia and carbon dioxide. At high temperatures, ammonia molecules may require more energy to overcome interactions with their surroundings and thus enter the hydrate cage, whereas carbon dioxide molecules may be more easily displaced due to their smaller molecular size and weaker interaction forces. When the temperature was further increased to 265 K, carbon dioxide outperformed ammonia substitution in terms of both the substitution rate and the number of methane molecules substituted as shown in Figure 20 [73]. This may be due to the significant increase in the diffusion rate of carbon dioxide molecules at high temperatures, which makes it easier for them to enter the hydrate cage and undergo replacement reactions with methane molecules. It was also noted that at a constant temperature, the change in pressure had some effect on the number of displaced methane molecules, but the overall trend did not change. This suggests that temperature is the main factor affecting the replacement efficiency during the replacement process, while pressure may indirectly affect the replacement efficiency by affecting the stability of the hydrate structure and the solubility of the gas.

### 4.3. Other Factors

Factors such as initial carbon dioxide concentration and salt ions play an important role in the replacement of methane in hydrates by carbon dioxide, and they influence the replacement efficiency to some extent. These factors do not exist in isolation, but may have complex interactions with each other. Therefore, in practical applications, various factors need to be considered to optimize the replacement conditions for efficient methane recovery and carbon dioxide sequestration.

**Figure 21:**
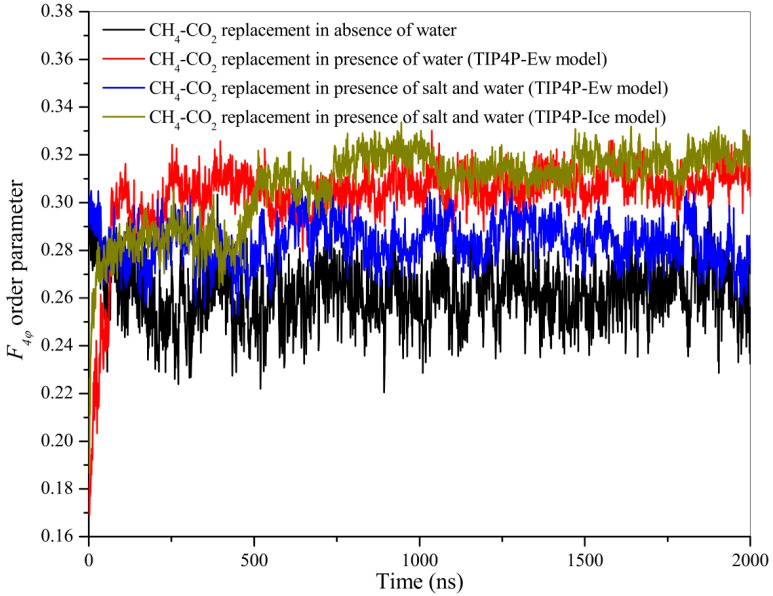
F4φ order parameters for different system substitutions at 260 K and 50 MPa (Palodkar, A.V. et al., 2022 [74]).

The study by Palodkar, A.V. et al. [74] sheds light on the effect of salt ions on the efficiency of the carbon dioxide replacement process with methane hydrate in different systems. In the absence of free water molecules in the system, the replacement reaction of carbon dioxide with methane hydrate proceeds directly without the interference of free water molecules. This system may be closer to the experimental environment under some specific conditions, but it is far from the complex situation in real geological reservoirs. When free water molecules are present in the system, carbon dioxide forms a new mixed hydrate layer on top of the existing hydrate phase during the replacement process. This finding is consistent with the findings of Bai et al. and suggests that free water molecules act as a hindrance in the replacement process because they combine with carbon dioxide to form a new hydrate phase, thus reducing the number of cage sites available for methane replacement. The presence of free water molecules and salt ions in the system significantly altered the efficiency of the substitution process. The addition of salt ions helped to inhibit the formation of mixed hydrates, possibly because the interaction between salt ions and water molecules altered the stability conditions of the hydrates. At the same time, salt ions may also reduce the stability of the hydrate by affecting its phase equilibrium, making it easier for methane to be released from the hydrate and replaced by carbon dioxide. The reason for this is that salt ions may reduce the opportunity for water and carbon dioxide molecules to form mixed hydrates by interacting with them, thus maintaining more cage sites for methane and carbon dioxide to be displaced. The presence of salt ions may also reduce the stability of hydrates by altering their phase equilibrium conditions, making it easier for methane molecules to escape from hydrate cages and thus increasing the replacement efficiency. In systems where free water molecules and salt ions are present, further reduction in the replacement pressure can further increase the replacement efficiency. This may be due to the fact that lowering the pressure favors the destabilization of the hydrate, making it easier for methane molecules to be released from the hydrate. At the same time, the lower pressure may also be favorable for the carbon dioxide molecules to diffuse into the hydrate more quickly for replacement with methane. The replacement process can be optimized and the replacement efficiency can be improved by rationally controlling conditions such as the concentration of free water molecules and salt ions in the system and the replacement pressure (Figure 21).

**Figure 22:**
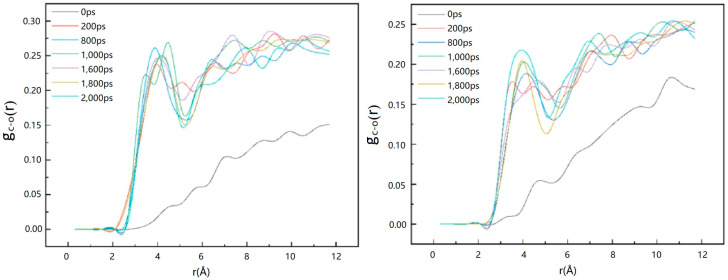
RDF of carbon dioxide hydrate with time in pure water and porous media systems (Zhang, X. et al., 2023 [75]).

In nature, natural gas hydrates are mainly distributed in the porous media sediments in the permafrost zone, which are mostly sandy soils with silica as the main component. This porous structure provides abundant interfaces and channels for gas and liquid diffusion, adsorption, and chemical reactions, and the effect of subfreezing porous media on carbon dioxide replacement in natural gas hydrates was thoroughly investigated in a study by Zhang, X. et al. [75]. Zhang et al. found that the silica in the porous media system has significant adsorption of carbon dioxide compared with the pure water system. This adsorption makes it easier for carbon dioxide gas to enter the hydrate layer and undergo a displacement reaction with the methane molecules therein to produce carbon dioxide hydrate. This adsorption not only increases the contact opportunity between carbon dioxide and methane hydrate, but also promotes the replacement reaction. As shown in Figure 22, the results indicate that carbon dioxide hydrate is more stable in the porous media system compared to the pure water system. This may be due to the additional support and stabilization provided by solid components such as silica in the porous media, which allows the carbon dioxide hydrate to remain stable for a longer period of time after formation. In addition, the tiny pores and channels in the porous media may also contribute to the formation and stabilization of carbon dioxide hydrate.

**Figure 23:**
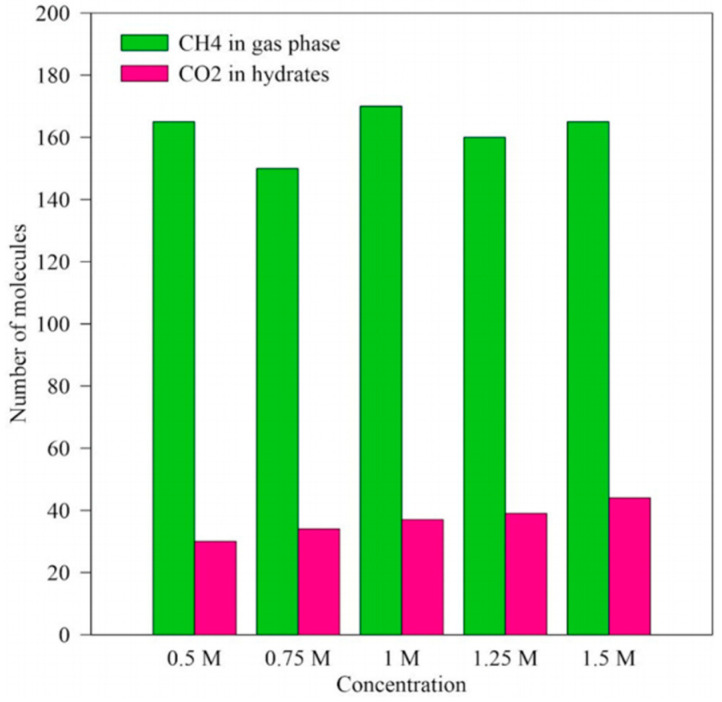
Changes in the number of methane and carbon dioxide molecules with initial carbon dioxide concentration (Gajanayake, S. et al., 2022 [67]).

The study by Gajanayake, S. et al. [67] offers a deeper understanding of the effect of initial carbon dioxide concentration on the replacement efficiency during the replacement of natural gas hydrates through MDS. This study was conducted under specific temperature and pressure conditions (280 K and 8 MPa) to ensure consistency of experimental conditions. A system containing 320 carbon dioxide molecules was used as the baseline concentration (defined as M), and four other systems with different concentrations of 160 (0.5 M), 240 (0.75 M), 400 (1.25 M), and 480 (1.5 M) carbon dioxide molecules were set up. The changes in the number of carbon dioxide molecules entering and stored in the hydrate structure at different initial carbon dioxide concentrations were observed and recorded by MDS. The results show that the replacement efficiency versus concentration is shown in Figure 23, and the number of carbon dioxide molecules stored in the hydrate structure increases with the increase in the initial carbon dioxide concentration. Increasing the initial carbon dioxide concentration in the free gas phase can significantly improve the replacement efficiency, allowing more carbon dioxide molecules to enter and be stably stored in the hydrate structure. From the perspective of carbon dioxide sequestration, increasing the initial carbon dioxide concentration means that more carbon dioxide can be sequestered in the same replacement time. This is important for reducing greenhouse gas emissions and combating climate change.

## 5. Conclusions

Molecular dynamics simulation is essential for understanding carbon dioxide applied for methane exploitation from hydrates, offering insights into the thermodynamic and kinetic mechanisms of the replacement process. By modeling molecular interactions, conditions such as temperature, pressure, and auxiliary gases can be optimized to improve recovery efficiency. This supports sustainable energy development by enabling efficient methane extraction while facilitating carbon sequestration. This dual benefit aligns with global goals of reducing emissions, enhancing energy security, and ensuring environmental and economic sustainability in hydrate exploitation.

A comprehensive review and analysis of numerous studies reveal that two mechanisms govern the carbon dioxide replacement of natural gas hydrates: guest molecular replacement and hydrate reconfiguration. The guest molecular replacement view involves a direct exchange between carbon dioxide and methane, possibly with partial cage structure disruption but without complete breakdown. This mechanism occurs under milder conditions, where carbon dioxide and methane molecules are similar in size, shape, and polarity, allowing easy exchange within the hydrate cage. The hydrate reconfiguration view suggests complete destruction of the hydrate cage under extreme conditions, leading to free water molecule production and subsequent recombination with carbon dioxide to form a new hydrate structure. Optimizing the carbon dioxide replacement process for methane hydrates involves determining optimal temperature and pressure conditions, adjusting initial gas phase concentration, controlling the presence of free water molecules, and selecting appropriate auxiliary gas types and ratios.

The future of molecular dynamics simulations for carbon dioxide applied for methane exploitation from hydrates is promising and multifaceted, leveraging machine learning for dynamic optimization, exploring alternative guest molecules, refining thermodynamic parameters, and ensuring environmental and economic feasibility. Advancements in computational power and algorithms will enable more accurate and complex simulations, providing deeper insights into the molecular mechanisms of gas exchange and hydrate stability to drive efficiency, sustainability, and innovation in methane recovery and energy production.

## Figures and Tables

**Table 1:** Number of water molecules in the hydrate phase at different temperatures (Zhang, K., 2019 [55]).

Temperature	Time (ns)	Hydrate Phase Surface	Hydrate Phase Interior
250 K	300	170.55 ± 4.07	184.45 ± 1.17
	2000	168.65 ± 2.85	184.4 ± 0.50
	300	166.20 ± 2.23	183.85 ± 0.32
260 K	2000	162.10 ± 5.20	184.25 ± 0.41
	300	163.90 ± 2.89	184.5 ± 0.46
270 K	300	170.55 ± 4.07	184.45 ± 1.17

**Table 2:** Carbon dioxide displacement rate and methane release rate at different temperatures (Zhang, K. et al., 2019 [55]).

Temperature	Time (ns)	Displacement Rate	Release Rate
250 K	300	0.057	0.088
	2000	0.0098	0.016
260 K	300	0.057	0.093
	2000	0.0095	0.020
270 K	300	0.075	0.127

## Data Availability

No new data were created.
